# Callus-Mediated High-Frequency Plant Regeneration, Phytochemical Profiling, Antioxidant Activity and Genetic Stability in *Ruta chalepensis* L.

**DOI:** 10.3390/plants11121614

**Published:** 2022-06-20

**Authors:** Ahmed A. Qahtan, Mohammad Faisal, Abdulrahman A. Alatar, Eslam M. Abdel-Salam

**Affiliations:** 1 Department of Botany and Microbiology, College of Science, King Saud University, P.O. Box 2455, Riyadh 11451, Saudi Arabia; aqahtan@ksu.edu.sa (A.A.Q.); aalatar@ksu.edu.sa (A.A.A.); 2 Plant Molecular Biology, Faculty of Biology, Ludwig-Maximilians-University Munich, 82152 Planegg, Germany; eabdelsalam@hotmail.com

**Keywords:** antioxidant activity, callus culture, in vitro regeneration, medicinal plants, phenolic compounds, plant growth regulators

## Abstract

Efficient methods for callus induction and the high-frequency plant regeneration of *Ruta chalepensis* L. were established, and the phytochemical potential and antioxidant activity of a donor plant, ex-vitro-established micropropagated plants, and callus were also studied. Yellowish-green callus was induced with a frequency of 97.8% from internode shoot segments of the donor plant growing in soil in the botanical garden cultured on Murashige and Skoog (MS) medium containing 10 μM 2,4-D (2,4-dichlorophenoxyacetic acid) and 1 μM BA (6-benzyladenine). Adventitious shoots were regenerated from the yellowish-green callus on MS medium containing 5.0 μM (BA) and 1.0 μM 1-naphthaleneacetic acid (NAA), with a regeneration frequency of 98.4% and a maximum of 54.6 shoots with an average length of 4.5 cm after 8 weeks. The regenerated shoots were rooted in a medium containing 1.0 μM IBA (indole-3-butyric acid) and successfully transferred to ex vitro conditions in pots containing normal garden soil, with a 95% survival rate. The amounts of alkaloids, phenolics, flavonoids, tannins, and antioxidant activity of the ex-vitro-established micropropagated plants were higher than in the donor plant and callus. The highest contents of hesperidin and rutin (93.3 and 55.9 µg/mg, respectively) were found in the ex-vitro-established micropropagated plants compared to those obtained from the donor plant (91.4 and 31.0 µg/mg, respectively) and callus (59.1 and 21.6 µg/mg, respectively). The genetic uniformity of the ex-vitro-established micropropagated plants was appraised by the ISSR markers and compared with the donor plant. This is the first report describing the callus-mediated plant regeneration, as well as the production of phenolic compounds and antioxidant activities in *R. chalepensis*, which might be a potential alternative technique for the mass propagation and synthesis of bioactive compounds such as hesperidin and rutin.

## 1. Introduction

Therapeutic plants include an abundance of secondary metabolites with significant medicinal properties. Bioactive compounds have strong therapeutic potential as antioxidative, anticancer, anti-inflammatory, and antimicrobial agents [1,2,3,4]. *Ruta chalepensis* L. is an herbaceous medicinal plant of the genus *Ruta*. It has traditionally been used in folk medicine for the treatment of rheumatism, neuralgia, epilepsy, headache, intestinal worms, convulsion, and menstrual disorders [5,6,7]. *R. chalepensis* is characterized by the presence of several bioactive constituents, e.g., flavonoids, phenols, alkaloids, coumarins, saponins, tannins, sterols, and triterpenes [8,9]. Different *R. chalepensis* extracts were studied by Kacem, et al. [10]. Among the studied extracts, ethanol and methanol extracts exhibited the highest total phenols, flavonoids, flavanols, and tannins contents. In ethanol extract, the most common flavonoid was rutin (6.11 mg/g DW). Loizzo, et al. [11] studied the phytochemicals in a methanol extract of *R. chalepensis* leaves. The methanol extract contained phenols and flavonoids. The analysis by HPLC revealed the presence of hesperidin, rutin, rhamnetin, chlorogenic acid, and quercetin. Abdel-Salam, et al. [12] quantified rutin in the methanolic extract of three different tissues of *R. chalepensis* using high-performance thin-layer chromatography (HPTLC). The leaves contained the highest amount of rutin (14.75 µg/mg), followed by the stems (6.58 µg/mg), and then the roots (2.80 µg/mg).

The escalating need for phytochemicals has refocused attention on tissue culture methods as a viable tool for enhancing the production of phytochemicals. When compared to traditional procedures, in vitro technologies have produced a huge number of medicinal plants and their metabolites in a very small space of time [13]. It was shown that *Nardostachys jatamansi* has total alkaloid, phenolic, flavonoid, and tannin content, as well as antioxidant activity, in the leaves and rootstocks of the donor plant, micropropagated plantlets, and callus [14]. Gupta, et al. [15] used three different solvents (methanol, 1:1 aqueous methanol, and acetone) to determine the content of phenols and flavonoids in micropropagated and wild plants of *Lysimachia laxa*. The findings revealed that the aqueous methanol extract of micropropagated plants had the higher concentrations of phenolics and flavonoids (20.47 GAE mg/g and 4.92 RE mg/g, respectively) when compared to wild plant extracts (11.54 GAE mg/g and 4.53 RE mg/g, respectively). When micropropagated plants were compared to the donor plant, the findings revealed that they had greater levels of antioxidant activity than the donor plant. According to Rajput and Agrawal [16], the micropropagated plants of *Atropa acuminata* had a much-increased content of flavonoids and phenolics when compared to the donor plant. The micropropagated plants also showed higher antioxidant activity than the donor plant. Additionally, Dilkalal, et al. [17] showed a significant production of phenolics and flavonoids in the indirect micropropagated plants of *Asystasia gangetica*, which was consistent with previous findings.

Compared with the conventional propagation techniques, micropropagation methods have the advantage of being able to achieve a high number of young plants in a short timeframe. Plant tissue culture has recently emerged as a strong tool for the multiplication and enhancement of several medicinal plants, including *Ruta*. Using leaf tissues from *R. graveolens*, Ahmad, et al. [18] established an effective callus induction and indirect plant regeneration method. Faisal, et al. [19] reported regeneration through the shoot tip meristems of *R. graveolens* by cytokinin–auxin synergism. In vitro multiplication of *R. chalepensis* through direct regeneration was recently reported by Qahtan, et al. [20].

The main objective of this study was to develop a plant propagation protocol via the indirect organogenesis of *R. chalepensis*. The phytochemical profile and antioxidant activity of the ex-vitro-established micropropagated plants were evaluated and compared to the callus and donor plant. Rutin and hesperidin were also quantified in the methanol extract of the plant materials using the high-performance liquid chromatography (HPLC) technique. In addition to this, the ex-vitro-established micropropagated plants of *R. chalepensis* were analyzed using ISSR markers in order to determine their genetic stability. 

## 2. Results 

### 2.1. Callus Induction

**Figure 1:**
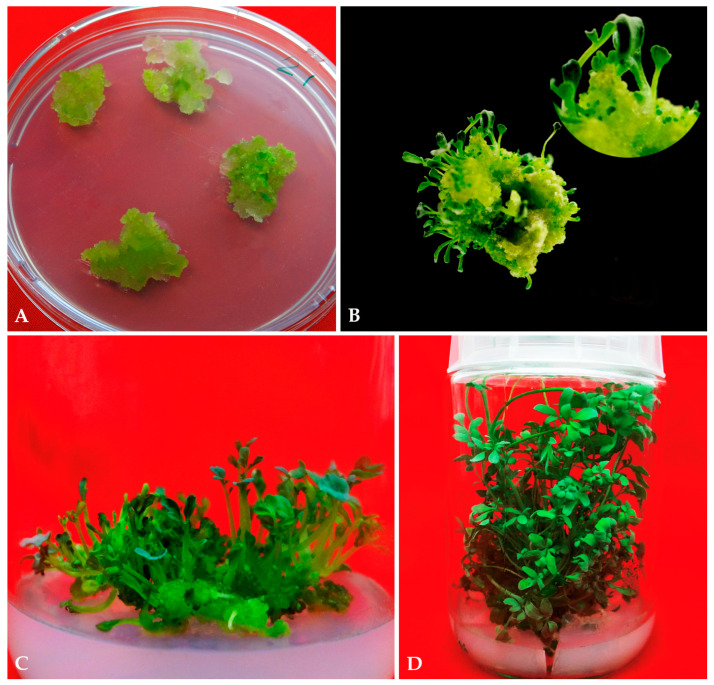
Callus induction and shoot bud induction and multiplication of *R. chalepensis*. (**A**). Callus induction in internodal explants on MS with 2,4-D (10.0 µM) + BA (1.0 µM) (**B**). Shoot bud initiation from callus on MS with BA (5.0 µM) + NAA (1.0 µM) (**C**). Multiple shoot induction from on MS with BA (5.0 µM) + NAA (1.0 µM) after four weeks of culture (**D**). Shoot proliferation on MS with BA (5.0 µM) + NAA (1.0 µM) after eight weeks of culture.

To produce callus, internode explants of the donor *R. chalepensis* plant growing in soil in the botanical garden were cultured on MS medium supplemented with various doses of 2,4-D (1.0–20.0 µM) either alone or in combination with different concentrations of BA or Kin (1.0 and 2.5 µM). The response rate and weight of the callus varied depending on the type of plant growth regulators (PGRs) applied. Callus formation did not occur without the PGRs. After 8–11 days of incubation, callogenesis was observed on the internode explants of *R. chalepensis*. The induction percentage, fresh weight, and dry weight of the callus varied depending on the PGRs used in the media. At a lower dose of 2,4-D (1 µM), 71.2% of the explants produced calli. The response rate, fresh weight, and dry weight of the callus gradually increased with increasing the concentration of 2,4-D up to 10 µM (Table 1). The combination of 2,4-D (10 µM) with cytokinins (1.0 and 2.5 of BA or Kin) had a significant effect on callus induction as it increased the response and weight of the callus. The color and texture of the calli that were induced differed depending on the growth regulator used. Depending on the media, two types of calli were observed: yellow and yellowish-green in color. The yellowish-green callus induced on media containing 10 μM 2,4-D and 1.0 μM BA was found to exhibit the faster growth, which was maintained by regular subculturing on to the same fresh media. After 6 weeks of culture on this medium, 97.8 percent of explants induced highly organogenic yellowish-green callus with the highest fresh weight of 672.5 mg per explant and dry weight of 68.6 mg per explant (Figure 1A, Table 1). 

### 2.2. Effect of Cytokinins on Shoot Bud Induction from Callus

Yellowish-green callus derived from internode explants induced on 10 μM 2,4-D and 1.0 μM BA was cut into 1000 mg of callus mass and subcultured on MS medium with varied concentrations (2.5–10 μM) of BA or Kin to generate shoot buds (Figure 1B). Generally, the shoot buds emerged in most treatments within three weeks of inoculation. A maximum shoot regeneration frequency of 85.5% with 30.4 shoots per callus mass and a mean length of shoot 3.9 cm was recorded on MS medium with 5.0 μM BA after 8 weeks of inoculation (Table 2). In contrast, the MS medium supplemented with Kin was shown to be less efficient than BA in the induction of shoots from the callus mass. Among the different Kin concentrations applied, a maximum number of 26.4 shoots per callus mass and an average length of shoot 3.5 cm was recorded on the MS medium supplemented with 5.0 μM Kin after 8 weeks of inoculation. 

### 2.3. Effect of Optimized Concentration of Cytokinins with Auxins for Shoot Regeneration 

**Figure 2:**
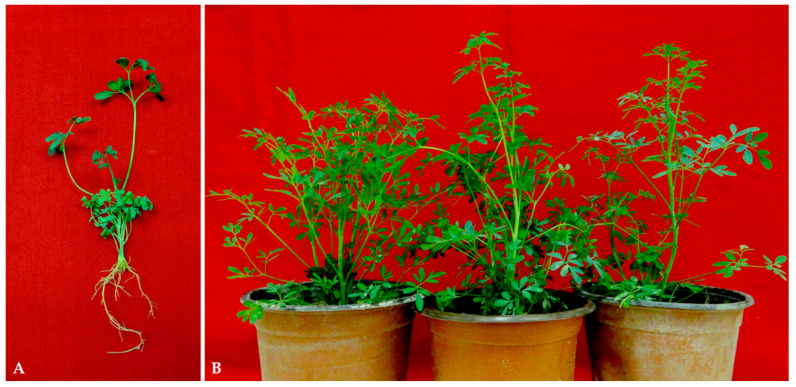
(**A**). In-vitro-rooted plants of *R. chalepensis* before transplantation (**B**). Ex-vitro-established micropropagated *R. chalepensis* plants after 4 months of transfer.

The combined effect of low concentrations (0.1–2.5 μM) of NAA, IAA, or IBA with an optimum concentration of BA and Kin (5.0 μM) for the induction of shoot buds from yellowish-green callus mass produced from internode explants on MS + 2,4-D (10 μM) + BA (1.0 μM) was also investigated. The shoot buds appeared in most treatments within three weeks of inoculation. The obtained findings were compared to a control of 5.0 µM BA. After 8 weeks of culture, the callus mass inoculated on MS medium supplemented with 5.0 µM BA and 1.0 µM NAA produced a maximum shoot induction (98.4%) and a maximum number of shoots (54.63 shoot per calls mass), with an average shoot length of 4.5 cm (Table 3 and Figure 1C,D). However, 5.0 µM BA in combination with 1.0 µM of IBA or IAA were less effective; they generated 43.6 and 39.4 shoots per callus mass, respectively. On the other hand, kin (5.0 µM) in combination with NAA, IAA, or IBA at 1.0 µM generated 41.8, 35.7, and 31.2 shoots per callus mass, respectively, after 8 weeks of culture (Table 3). The well-developed shoots of *R. chalepensis* regenerated on MS + BA (5.0 µM) + NAA (1.0 µM) were rooted in a medium containing 1.0 μM IBA (Figure 2A). In ex vitro conditions, the rooted plantlets were effectively hardened off and continued to grow normally (Figure 2B), resulting in a survival rate of 95%.

### 2.4. Phytochemical Screening

The contents of some secondary metabolites, such as polyphenols, flavonoids, alkaloids, and tannins, in the ex-vitro-established micropropagated plants were determined and compared with the donor plant and callus. The amounts of alkaloids, phenolics, flavonoids, and tannins in the ex-vitro-established micropropagated plants were higher than in the donor plant and callus (Table 4). The amounts of alkaloids present in 0.5 g of the plant samples was 10.7% in the ex-vitro-established micropropagated plants, 8.9% in the donor plant, and 7.9% in the callus. The methanolic extract of the ex-vitro-established micropropagated plants showed higher values for phenolic, flavonoid, and tannin contents (143.9 mg GAE/g DW, 135.8 mg QE/g DW, and 54.3 mg TAE/g DW, respectively), followed by the donor plant and callus, respectively (Table 4). 

### 2.5. Antioxidant and Free Radical Scavenging Activity

The antioxidant activities of the methanolic extracts of the *R. chalepensis* the donor plant growing in soil in the botanical garden, ex-vitro-established micropropagated plants, and callus obtained on MS + 2,4-D (10 µM) + BA (1.0 µM) were determined using free radical scavenging activity by the 2,2-diphenyl-1-picrylhydrazyl (DPPH) method. In general, the concentration increments in all the extracts resulted in higher scavenging activity against the DPPH radical, reaching its maximum activity at 600 µg/mL, then started to decrease (Table 5). The results indicated that the methanolic extract of the in vitro plants showed the highest percentage inhibition value of DPPH radical at 600 µg/mL (91.2%) compared to the donor plant (88.9%) and callus (72.9%). 

### 2.6. Gas Chromatography–Mass Spectrometry (GC–MS) Analysis of Extracts

**Figure 3:**
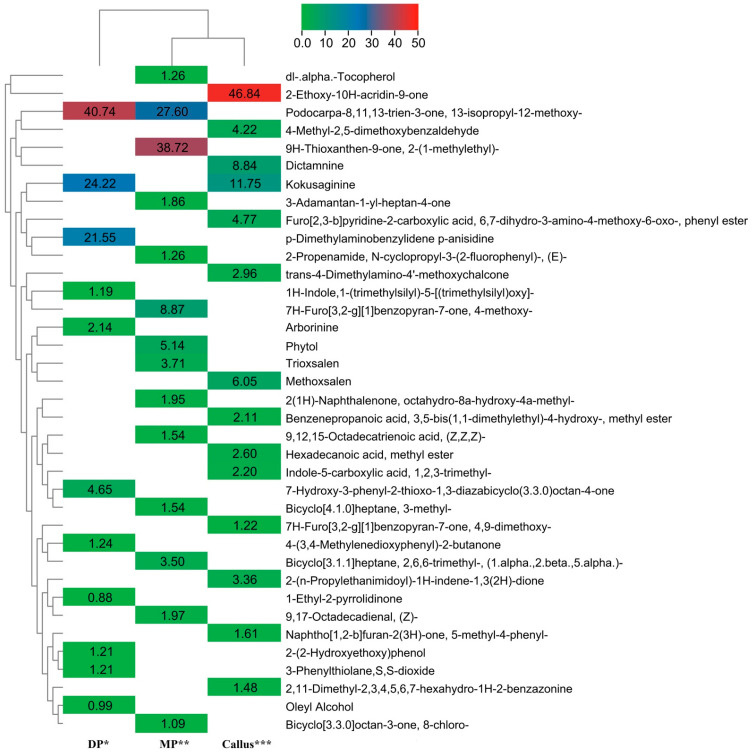
Cluster heatmap analysis based on the relative levels of phytochemicals measured by GC–MS in *R. chalepensis.* The magnitude and direction of the correlations are shown by the colors in the matrix boxes. * DP = donor plant growing in soil in the botanical garden; ** MP = ex-vitro-established micropropagated plants; *** callus = callus obtained on MS + 2,4-D (10 µM) + BA(1.0 µM).

The methanolic extract of the ex-vitro-established micropropagated plants and callus contained fourteen phytocompounds, while the donor plant growing in soil in the botanical garden contained eleven compounds. The names of the compounds with their retention times (RT), area (%), molecular weight, and structural formula are presented in Table 6. Indeed, 2-Ethoxy-10H-acridin-9-one (46.8%), Podocarpa-8,11,13-trien-3-one, 13-isopropyl-12-methoxy- (40.4%), and 9H-Thioxanthen-9-one, 2-(1-methylethyl)- (38.7%), were found in high percentages in the callus, donor plant, and ex-vitro-established micropropagated plants, respectively. The main phytochemicals analyzed in the extract were Arborinine, 9,17-Octadecadienal, (Z)-, dl-.alpha.-Tocopherol, Kokusaginine, Methoxsalen, Oleyl Alcohol, Phytol, Dictamnine, and Hexadecanoic acid methyl ester (Table 6 & Figure 3).

### 2.7. Rutin and Hesperidin Quantitation

In this present study, we examined the contents of rutin and hesperidin in the methanolic extract of ex-vitro-established micropropagated plants, a donor plant growing in soil in the botanical garden, and callus obtained on MS + 2,4-D (10 µM) + BA(1.0 µM) using HPLC. All the plant extracts contained rutin and hesperidin but in different concentrations. The highest content of hesperidin and rutin (93.3 and 55.9 µg/mg, respectively) was found in the ex-vitro-established micropropagated plants compared to that obtained from the donor plant (91.4 and 31.0 µg/mg, respectively) and callus (59.1 and 21.6 µg/mg, respectively) (Table 7).

### 2.8. Genetic Fidelity Using ISSR Marker

**Figure 4:**
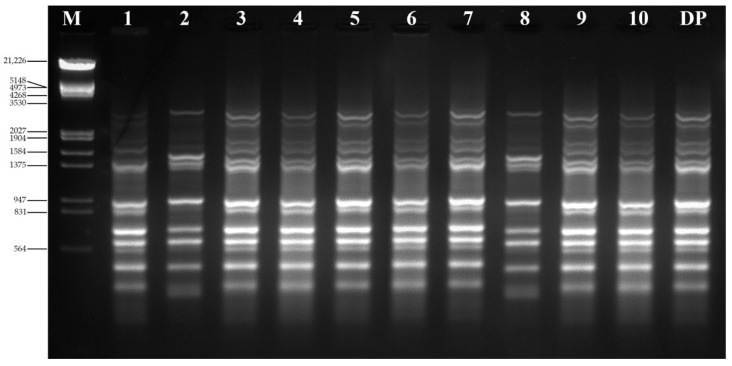
Representative ISSR profiles of *Ruta chalepensis* using primer UBC-827. Lane M—lambda DNA/EcoRI+HindIII marker; lanes 1–10 randomly selected ex-vitro-established micropropagated plants; lane DP—donor plant growing in soil in the botanical garden.

To examine the genetic uniformity of the micropropagated plants using ISSR analysis, ten ISSR primers were tested for the initial screening. All the primers produced clear, scorable, and reproducible bands after PCR amplification. These primers developed 131 scorable bands with an average 13.1 bands per primer (Table 8). The ISSR primer UBC-827 generated the maximum number of bands (16), whereas UBC-834 generated the lesser number of bands (9). The ISSR analysis revealed that all of the bands obtained in both the ex-vitro-established micropropagated plants and donor plant were 100% similar as well as monomorphic (Figure 4).

## 3. Discussion

The efficacy of callus induction is contingent upon a number of factors, including the type of explant used, as well as the concentration and combination of PGRs used [21]. As a result of this study, callus was successfully produced from the internode explant of the donor plant cultivated on MS medium supplemented with various doses of 2,4-D (1–20 μM) either alone or in combination with BA or Kin (1 and 2.5 μM), indicating that the culture medium significantly influenced the callusing response. Only 71.2 percent of the internodal cultures induced callus at a dose of 1.0 μM 2,4-D, but the frequency of callus induction increased to 97.8% with an increasing concentration of 2,4-D to 10 μM. Indeed, 2,4-D has been shown to be effective in inducing callus in a variety of plant species [22,23,24,25]. The varying response in callus induction and morphology may be explained by PGRs’ function in managing the cell cycle; they regulate key enzymes involved in the cell cycle, and the exogenous application of PGR modifies the levels of other endogenous hormones, resulting in variance in the developmental process and variation in the dissipation of the plant cell wall [26]. Moreover, 2,4-D plays an important role in cell division and elongation, as well as regulates cell development [27]. When lower amounts of cytokinins (BA) were introduced to MS media enriched with 2,4-D, the growth rate and callus mass increased. The source, age, and amount of endogenous phytohormones in the explant, as well as the type, concentration, and action mode of the auxin and cytokinin hormone used, may be very important for the formation of calli [28,29]. Similarly, in *Asparagus officinalis* [30], *Betula platyphylla* [31], *Coriandrum sativum* [21], and *Notopterygium incisum* [32], the addition of a low concentration of BA to an MS medium supplemented with 2,4-D induced efficient callus formation.

Plant growth regulators are important in initiating and developing shoots from callus. In our study, it was shown that low doses of BA (5.0 µM) had the highest probability of generating shoots from callus, with a greater response and number of induced shoots than higher doses (>5.0 μM). High dosages of cytokinin have been demonstrated to have deleterious effects on the multiplication of shoots in a range of plant species [20,33]. In a number of species, including *Ruta graveolens* [18], *Citrus jambhiri* [34], *Notopterygium incisum* [32], and *Mondia whitei* [35], BA has been shown to be highly effective in inducing shoot buds from callus mass. However, synergistic interactions between BA (5.0 μM) and NAA (1.0 μM) resulted in a greater response in the induction and multiplication of shoot buds from the callus mass. The optimal amount of cytokinins and auxins in the medium is critical for the induction and multiplication of shoot buds since it varies depending on the kind of explant, the cultivar, and the type of plant [36]. In their study, Wang, et al. [37] found that excessive cytokinins and auxins adversely influence plant regeneration. The effectiveness of the micro-propagation process depends on the balance between the levels of cytokinins and auxins, which synergistically affect the cell division and, hence, produce a larger number of shoots per callus mass than cytokinin alone [38,39]. In several plant species, a combination of NAA and BA was shown to be efficient for the induction and elongation of shoots from callus mass. The use of MS enhanced with BA and NAA was shown to be superior to other plant growth regulators for indirect regeneration from callus produced from leaf explants in *Ruta graveolens* [18]. Using a combination of BA and NAA, Bakhtiar, et al. [40] demonstrated the production of the greatest number of shoots from calli of *Thymus persicus*. MS media supplemented with (1 mg/L) BA and (0.05 mg/L) NAA produced 14.7 shoots per callus from root explants in *Aspilia africana*, with an 86.7% regeneration frequency [33]. Phytochemicals have a range of biological activities, such as anti-cancer, antimicrobial, anti-inflammatory, antioxidant, diabetes, and cardiovascular regulatory effects [2,14,41]. In this study, the contents of phenols, flavonoids, tannins, and alkaloids were higher in the ex-vitro-established micropropagated plants compared to the donor plant and callus. This may be explained by the fact that in vitro culture systems and PGRs enhance or accumulate several of the bioactive compounds. In vitro culture has been shown to increase the amounts of phytochemical compounds in many medicinal plants [15,16,17,42,43,44], which is consistent with our findings. In the *Aloe arborescens*, media supplemented with PGRs increased the quantity of total phenolics, tannins, and flavonoids compared to media without PGRs during micropropagation [45]. Thiruvengadam and Chung [44] mentioned that cytokinins significantly increased the total content of polyphenols and flavonoids in the micropropagated plants compared to wild plants of *Cucumis anguria*. Natural antioxidants are dietary additives that minimize the oxidation of minerals in foodstuffs, as well as the detrimental consequences of reactive oxygen species (ROS)-induced oxidative stress. There is a statistically significant correlation between disease etiology and levels of oxidative stress. Antioxidants are able to minimize and prevent damage from free-radical reactions because of their potential to contribute electrons that neutralize the radical production [46,47,48]. Many plants produce secondary metabolites, such as flavonoids and polyphenols, that serve as antioxidants and play a crucial role in a variety of biological processes [49,50,51]. Plants and natural products are, thus, a significant source of antioxidants, which may eliminate free radical damage and protect the organism from the harmful effects of excessive oxidative stress. As a result of our research, we found that methanolic extracts had varying levels of antioxidant activity. The high levels of phytochemical components in the methanolic extracts may account for the extracts’ remarkable DPPH radical scavenging activity. Because of their redox characteristics and their capacity to form complexes with proteins and bacterial membranes, these chemicals are particularly efficient in scavenging free radicals [52]. These chemicals also have a high ability to get rid of ROS, such as hypochlorous acid, hydroxyl radical, peroxynitrite, and superoxide an-ion, which can affect plant’s metabolism by damaging proteins, lipids, and nucleic acids through oxidation [53]. Similarly, the micropropagated plants of *R. graveolens* [54], *Ceropegia santapaui* [55], *Cucumis anguria* [44], *Dendrobium thyrsiflorum* [56], *Nardostachys jatamansi* [14], and *Lysimachia laxa* [15] had higher antioxidant activity than the donor plant. The GC–MS method was used to identify secondary metabolites, such as alkaloids, sterols, polyphenols, terpenoids, and other chemicals, in plant extracts. Akkari, et al. [57], Jaradat, Adwan, K’aibni, Zaid, Shtaya, Shraim, and Assali [7], and Barbouchi, et al. [58] have all described the phytochemical analysis of *R. chalepensis* using the GC–MS method. However, this is the first report to use GC–MS to assess chemical homogeneity and to investigate the pharmacologically significant chemicals in a donor plant, ex-vitro-established micropropagated plants, and callus in *R. chalepensis*. A greater number of phytochemical components were found in the methanolic extracts from ex-vitro-established micropropagated plants and callus compared to those found in the donor plant. Plant tissue culture offers an excellent technological foundation for the quick and efficient production of phytochemical substances for commercial purposes [13,59]. It is likely that ex-vitro-established micropropagated plants’ increased production of phytochemical substances is attributable to a variety of factors, including the media they are growing in, the explants they are using, their genotype, and even the PRGs they have added to the medium [60,61]. Our findings are in line with previous studies that found that micropropagated plants had more phytochemicals than their donor plants, such as *Clerodendrum thomsoniae* [62], *Hemidesmus indicus* [63], *Tecoma stans* [64], *Hildegardia populifolia* [65], and *Diplocyclos palmatus* [61].

Rutin, a major flavonoid in the *Ruta*, possesses anti-inflammatory [66], antiallergic [67], anticancer [68], antimicrobial [69], and anticonvulsant [70] properties. Hesperidin is a bioactive flavonoid glycoside abundantly found in the Rutaceae. It has many pharmacological effects, including anti-cancer, anti-inflammatory [71], antidiabetic, antioxidant [72], and can lower blood pressure and cholesterol levels [73]. In this study, we examined the contents of rutin and hesperidin in the methanolic extract of callus, ex-vitro-established micropropagated plants, and a donor plant using HPLC. It was found that the donor plant, ex-vitro-established micropropagated plants, and callus cultures produce rutin and hesperidin. The concentrations of rutin and hesperidin in the extracts, on the other hand, were significantly different. In comparison to the donor plant and callus, the ex-vitro-established micropropagated plantlets had the greatest concentrations of rutin and hesperidin (55.9 and 93.3 g/mg, respectively). The possible reasons for the manifold enhancement of bioactive compounds in the micropropagated plants compared to the wild plants could be influenced by (i) stress induction during ex vitro hardening affecting the biosynthetic pathways, (ii) availability of adequate nutrients, and (iii) the presence of other physical, chemical, and biological elicitors. Several authors have reported that micropropagated plants contain increased bioactive components compared to wild plants [74,75]. The adventitious roots of *Morus alba* that were cultivated on MS medium with IAA (5 mg/L) and ammonium/nitrate (34/66 mM) could produce the greatest rutin concentration (260 g/g) [76]. In *Cucumis sativus*, the micropropagated plantlets of the Waffir cultivar produced higher levels of rutin and hesperidin (1.876 and 9.737 mg/100 g DW, respectively) than in vivo plants (0.586 and 4.440 mg/100 g DW, respectively) and callus (not detected and 4.858 mg/100 g DW, respectively) [77].

True-to-type genetic fidelity is one of the most important prerequisites in the micropropagation of any plant species. It is plausible that somaclonal variations exist in micropropagated plantlets, particularly in plants generated from callus [78]. As a consequence, it is now important to execute genetic stability tests of indirectly micropropagated plantlets using methods based on DNA analysis in order to confirm that they are genetically identical to the donor plant [79]. There is a plethora of PCR-based DNA markers that may be utilized to establish genetic stability. In our study, ISSR primers revealed that all the ex-vitro-established micropropagated plants were genetically identical to the donor plant, indicating that genetic homogeneity had been maintained across the regenerants. Similar studies on the genetic stability of regenerated plants using the ISSR molecular marker have been reported before in *Bacopa monnieri* [80], *Ruta graveolens* [19], *Morus alba* [81], *Rauwolfia tetraphylla* [82], *Ficus carica* [83], *Flemingia macrophylla* [84], *Solanum khasianum* [85], and *Thunbergia coccinea* [86].

## 4. Materials and Methods

### 4.1. Plant Material and Surface Sterilization

The young internode pieces of *R. chalepensis* were collected from a plant (donor plant) growing in the Botany and Microbiology Department of the King Saud University in Riyadh, Saudi Arabia. The explants were washed by laboratory tap water for 15 min, then treated for 6 min in a 5% (*v*/*v*) liquid detergent, followed by 3–5 rinses with sterile ultrapure (Milli-Q) water to remove any detergent residue. In a laminar flow hood (ESCO Labculture^®^ Class II Type A2 Biological Safety Cabinet, Esco Micro Pte. Ltd., Singapore), these explants were surface-sterilized with 0.1% mercuric chloride (HgCl_2_) for three minutes. Finally, the explants were washed 4–5 times with autoclaved ultrapure (Milli-Q) water and cut into pieces 0.5–0.7 cm in length before being placed to the callus induction medium as described below.

### 4.2. Culture Conditions and Callus Induction

Callus was induced from sterile internode segments of the above donor plant of *R. chalepensis* on Murashige and Skoogs’s (MS) [87] medium supplemented with sucrose (3% *w/v*) and agar (0.8% *w/v*) with 2,4-D at various doses (1.0, 2.5, 5.0, 10, and 20 μM) alone or in combination with BA or Kin at various doses (1.0 and 2.5 μM). After two weeks of culture, the callus was subcultured into the same fresh medium. After six weeks, the percentage of callus induction was determined, as well as the fresh and dried weights of calli, which were then transferred to shoot induction media.

### 4.3. Shoot Multiplication

Adventitious shoot regeneration was achieved by culturing the yellowish-green callus (about 1 g) induced on MS + 2,4-D (10 μM) + BA (1.0 μM) from internode segments of the above donor plant on MS medium containing 3% sucrose, 0.8% agar, and different concentrations (1.0–10% M) of 6-benzyladenine (BA) or Kinetin (Kin), either alone or in combination with different concentrations (0.5–2.0 μM) of auxins, i.e., indole 3-butyric acid (IBA), 1-naphthaleneacetic acid (NAA), or indole-3-acetic acid (IAA). After four weeks, all of the cultures were subcultured in the same fresh media. After eight weeks of culture, we recorded the % response and the mean number of shoots with the average shoot length per explant. The in vitro regenerated microshoots of *R. chalepensis* were rooted in a growth medium containing 0.5 μM IBA, and the rooted plantlets were hardened off and successfully grown out of an ex-vitro environment.

### 4.4. Rooting and Acclimatization

For rooting, *R. chalepensis* regenerated shoots that had grown on media with BA (5.0 μM) and NAA (1.0 μM) were moved to MS medium with 0.5 μM IBA. After 4 weeks, the microshoots with roots were carefully taken out of the culture tube and washed with water in order to remove any agar that was still there. After that, they were planted in plastic pots containing potting soil (Planta Guard, GRUBE, Bispingen, Germany) and covered with transparent plastic covers. The plantlets were kept in a growth environment under a 16/8 h (day/night) photoperiod with a photon flux density of 50–60 μmol m^−2^ s^−1^ provided by cool fluorescent lamps (40 W, Osram GmbH, Munich, Germany). After one month, acclimated micropropagated plants were successfully moved into pots with normal garden soil and kept in a greenhouse with natural day light.

### 4.5. Phytochemical Analysis

#### 4.5.1. Plant Material and Extract Preparation

Fresh leaves of the donor plant growing in soil in the botanical garden, ex-vitro-established micropropagated plant, and callus induced on MS + 2,4-D (10 µM) + BA (1.0 µM) were dried in an oven at 50 °C for 24 h and then ground into a fine powder. 1.0 g of the powdered material was macerated with 50 mL of methanol and kept at room temperature for 24 h with periodic shaking. Then, the mixture was filtrated through Whatman No. 1 filter papers. The extraction process was repeated three times. The solvent was dried using a rotary evaporator (IKA^®^-Werke GmbH & Co. KG, Staufen, Germany).

#### 4.5.2. Determination of Total Phenolics

The total phenolic contents of *R. chalepensis* plant extracts were determined using the technique established by Singleton, et al. [88], with some modifications. Gallic acid solutions (25–150 µg/mL) were used to generate a standard curve. 0.1 mL of plant extract (1 mg/mL) or gallic acid, 1.5 mL of ultrapure (Milli-Q) water, and 0.1 mL of Folin-Ciocalteu reagent were mixed and allowed to sit for 8 min. A volume of 0.3 mL of 20% sodium carbonate solution was added and mixed well using a vortex. The mixture was incubated in darkness for 2 h. The absorbance of the resulting blue color was measured at 765 nm using a UV–visible spectrophotometer. The total phenolic content of the extracts was quantified as gallic acid equivalent (mg/g DW) using the equation based on the calibration curve (*y =* 0.005 − *x* − 0.0088), where *y* was the absorbance and *x* was the gallic acid equivalent concentration (mg/g).

#### 4.5.3. Determination of Total Flavonoids

Method described by Ordoñez, et al. [89] was used to determine the total flavonoids in *R. chalepensis* plant extracts. 1.0 mL of plant extract (1 mg/mL) and 1.0 mL of 2% AlCl_3_ water solution were mixed. After an hour of incubation at room temperature, absorbance was measured at 420 nm. The standard solution was prepared using a quercetin solution (50–800 μg/mL) to generate a standard curve (R^2^ = 0.9996). The flavonoids in the extracts were expressed as quercetin (mg/g DW) using the calibration curve equation (*y* = 0.0011*x* + 0.0928), where *y* was the absorbance and *x* was the quercetin equivalent concentration (mg/g).

#### 4.5.4. Determination of Total Tannins

The total tannins in *R. chalepensis* plant extracts were determined using the method reported by Chandran and Indira [90], with some modifications. Tannic acid solutions (25–200 µg/mL) were used to generate a standard curve. Approximately 100 µL of the extract (1 mg/mL) was mixed with 200 µL of Folin-Ciocalteu reagent, 1.5 mL of ultrapure water, and 200 µL of 35% sodium carbonate solution. A thorough shaking of the mixture followed, accompanied by an incubation period of 30 min at room temperature. A UV–visible spectrophotometer was used to measure the absorbance of the ensuing blue color at 700 nm. On the basis of the calibration curve, the total tannin content in the extracts was represented as tannic acid equivalent (mg/g DW) using the following equation (*y* = 0.0052*x* + 0.0021), where *y* denoted absorbance and *x* denoted the tannic acid equivalent concentration (mg/g).

#### 4.5.5. Determination of Total Alkaloids

The total alkaloids content in the plant extracts was measured as described by Harborne [91], with some modifications. 50 mL of 20% glacial acetic acid in ethanol was added to 500 mg of the powdered plant sample in a 100 mL flask and then left on the shaker overnight for extraction and then filtered. The extract was incubated in a water bath at 100 °C for ½ h. Thereafter, concentrated ammonium hydroxide (NH_3_OH) was slowly added into the extract until some precipitation was obtained. After disposal of the supernatant, the precipitate was washed with dilute NH_3_OH (1%) and then filtered through Whatman No. 1 filter papers. After drying in an oven, the dried papers with residue were weighed. The alkaloid content was then calculated using the equation:

$$Alkaloid\%=\frac{\mathrm{Final}\;\mathrm{weight}\;\mathrm{of}\;\mathrm{the}\;\mathrm{sample}\;}{\mathrm{Initial}\;\mathrm{weight}\;\mathrm{of}\;\mathrm{the}\;\mathrm{sample}}\times 100$$

#### 4.5.6. Determination of Antioxidant and Free Radical Scavenging Activity

The antioxidant activities of *R. chalepensis* donor plant growing in soil in the botanical garden, ex-vitro-established micropropagated plant, and the callus induced on MS + 2,4-D (10 µM) + BA (1.0 µM) were determined by the 2,2-diphenyl-1-picrylhydrazyl (DPPH) free radical scavenging assay [92]. In this experiment, 1 mL of the extract was combined with 1 mL of 0.135 mM DPPH at various concentrations (200–1000 g/mL). For 40 min, the mixture was kept at room temperature in the dark and gently stirred. Ascorbic acid was used as a positive control. At 517 nm, the absorbance of the samples and the control solutions were examined and % of DPPH scavenging activity of the extract was calculated using the following equation:

DPPH scavenging activity (%) = [(Abs control − Abs sample)/Abs control] × 100, where Abs control is the absorbance of DPPH + methanol and Abs sample is the absorbance of DPPH radical + sample.

### 4.6. Gas Chromatography–Mass Spectrometry (GC–MS)

The phytochemical compounds in *R. chalepensis* extracts of the donor plant growing in soil in the botanical garden, ex-vitro-established micropropagated plant, and the callus obtained from MS + 2,4-D (10 µM) + BA (1.0 µM) were identified using a GC-MS (Turbomass, PerkinElmer, Inc., Waltham, MA, USA). The temperature of program 4 was set to 40 °C, followed by a 2 min hold, and then was raised to 200 °C at a rate of 5 °C min^−1^, which was put on hold for 2 min. From 200 °C, again, the temperature was raised by 5 °C min^−1^ to 300 °C and held for another 2 min. The chemical compositions of extracts were determined by comparing the acquired mass spectra to those from the National Institute of Standard and Technology and WILEY Spectral libraries. The mass spectra of compounds were also compared to those of comparable compounds included in the Adams Library (183) and the Wiley GC/MS Library (184). Compounds were characterized by comparing their RT (retention time) to genuine reference standards under the identical conditions described above.

### 4.7. High-Performance Liquid Chromatography (HPLC) Analysis

Agilent liquid chromatographic system- USA with the column SB-C18 (1.8 μm, 4.6 × 150 mm) was employed for separation and quantification of rutin and hesperidin with authentic standards, which were purchased from Sigma Company (Sigma-Aldrich Co., Saint Louis, MO, USA).

#### 4.7.1. Rutin Quantification

For separation and quantification of rutin, from plant material, the mobile phase consisted of acetonitrile solution (A) and methanol (B) (40:60). The flow rate was 0.500 mL/min, and the injection volume of samples was 1 µL. The column temperature was adjusted to be 25 °C, while the wavelength was 278 nm. Rutin in plant materials was estimated from the linear equation (*y =* 1.1195*x* + 22.934) of a standard curve prepared with rutin standard (0.25, 0.5, and 1 mg/mL).

#### 4.7.2. Hesperidin Quantification

For hesperidin separation and quantification from plant material, the mobile phase was composed of acetonitrile (A) and methanol (B) (40: 60) (*v*/*v*). The injection volume of samples was 1µL with 0.800 mL/min flow rate. The column temperature was adjusted to be 27 °C. Chromatogram was acquired at 274 nm wavelength. Hesperidin contents in plant samples were determined by the retention time spiking with the authentic standard under the same settings, while the quantification of hesperidin was conducted from the linear equation (*y =* 0.6022*x* + 21.037) of a standard curve prepared with hesperidin 0.25, 0.5, and 1 mg/mL).

### 4.8. DNA Extraction and Amplification by ISSR Primers

Doyle and Doyle [93] method was used to isolate and purify DNA from fresh leaves (approximately 0.25 g) of the donor plant growing in soil in the botanical garden and ex-vitro-established micropropagated plant. Using a Nanodrop spectrophotometer (Nanodrop 2000, Thermo Scientific, Waltham, MA, USA), the purity and concentration of DNA was determined. Ten ISSR primers (UBC, Vancouver, BC, Canada) were used to check the genetic fidelity of micropropagated plant. PCR reactions were performed in a 25 μL reaction volume containing 1.5 µL genomic DNA (50 ng/μL), 1.5 µL primer, 12.5 µL of PCR master mix (GoTaq^®^ Green Master Mix, 2X, Promega, Madison, WI, USA), and 9.5 µL ultrapure (Milli-Q) water. PCR reaction was carried out in a Thermal Cycler (Bio-Rad, Hercules, CA, USA) with the following conditions: initial denaturation for 5 min at 94 °C, 34 cycles of denaturation step at 94 °C (45 s), primer annealing 46–55 °C (30 s), extension at 72 °C (90 s), and a final extension step for 5 min at 72 °C. To avoid false results and to ensure that the ISSR markers performed the same way each time, all the experiments were repeated three times. PCR products were photographed by a gel documentation system (G: BOX F3, Syngene, Cambridge, UK) after being separated by electrophoresis on a 1.5% (*w*/*v*) agarose gel in 1X Tris acetic acid EDTA (TAE) buffer at 75 V.

### 4.9. Statistical Analysis

Data for in vitro callus induction and shoot multiplication were collected after six and eight weeks, respectively. The experiment was performed three times, with 20 repetitions for each treatment. Three replicates were used for phytochemical assay and antioxidant activity. All the collected data were subjected to analysis of variance (ANOVA), and Duncan’s multiple range tests were used to determine the significant differences (*p* ≤ 0.05) between the treatment values by IBM-SPSS software version 26.0.

## 5. Conclusions

For the first time, the high-frequency indirect regeneration from the internode explant of *R. chalepensis* was investigated, along with its phytochemical potential and the antioxidant activities of the methanolic extracts of callus, ex-vitro-established micropropagated plants, and a donor plant growing in soil in the botanical garden. Callus and multiple shoots from internode explants have been successfully achieved utilizing MS and a distinct combination of PGRs. The callus induction was achieved with the best PGRs combination of 2,4-D (10 μM) and BA (1 μM), whereas the shoot multiplication was achieved with the best PGR combination of BA (5 μM) and NAA (1 μM). A considerable number of phytochemical substances were found in the ex-vitro-established micropropagated plants, donor plants, and callus, as well as strong antioxidant activity in the callus. The ex-vitro-established micropropagated plants, on the other hand, had high levels of phytochemical compounds and antioxidant activities. The effectiveness of the *R. chalepensis* micropropagation system in the synthesis of rutin and hesperidin was demonstrated in this work, indicating that it might be a useful alternative approach for the large-scale production of bioactive chemicals.

## Figures and Tables

**Table 1:** Effect of 2,4-D alone or in combination with BA or Kin on callus induction, fresh, and dry weight of callus from internodes after 6 weeks of culture.

PGRs (μM)	Response %	FW (mg)	DW (mg)
0.0	0.0 ± 0.0	0.0 ± 0.0	0.0 ± 0.0
1.0 µM 2,4-D	71.23 ± 0.74 ^f^	92.11 ± 1.66 ^i^	9.66 ± 0.21 ^h^
2.5 µM 2,4-D	79.61 ± 0.75 ^e^	192.15 ± 0.99 ^g^	18.82 ± 0.44 ^g^
5.0 µM 2,4-D	91.23 ± 0.82 ^c^	285.35 ± 1.52 ^f^	26.12 ± 0.62 ^f^
10 µM 2,4-D	94.46 ± 0.83 ^b^	391.15 ± 2.24 ^e^	37.45 ± 0.69 ^e^
20 µM 2,4-D	79.20 ± 1.69 ^e^	178.12 ± 4.4 ^h^	16.43 ± 0.47 ^h^
10 µM 2,4-D + 1.0 µM BA	97.82 ± 0.86 ^a^	672.52 ± 0.23 ^a^	68.61 ± 0.51 ^a^
10 µM 2,4-D + 2.5 µM BA	95.66 ± 1.36 ^b^	484.03 ± 1.11 ^b^	49.24 ± 0.59 ^b^
10 µM 2,4-D + 1.0 µM Kin	81.03 ± 0.89 ^e^	372.12 ± 1.94 ^d^	39.02 ± 0.99 ^d^
10 µM 2,4-D + 2.5 µM Kin	84.65 ± 1.44 ^d^	429.76 ± 2.93 ^c^	45.14 ± 1.36 ^c^

Values are means ± SEM, n = 20 per treatment group. Means in a column without a common superscript letter differ (*p* < 0.05), as analyzed by one-way ANOVA and the Duncan test.

**Table 2:** Effect of cytokinins on shoot multiplication in callus mass obtained from internodal explants after 8 weeks of culture.

Cytokinins (μM)		Response %	Mean Number of Shoots	Mean Shoot Length (cm)
BA	Kin			
0.0	0.0	0.0 ± 0.0 ^h^	0.0 ± 0.0 ^h^	0.0 ± 0.0 ^h^
1.0	-	73.26 ± 0.92 ^d^	21.83 ± 0.58 ^fg^	2.28 ± 0.09 ^ef^
2.5	-	79.43 ± 0.79 ^b^	25.07 ± 0.83 ^cd^	2.86 ± 0.12 ^c^
5.0	-	85.54 ± 0.58 ^a^	30.43 ± 0.51 ^a^	3.98 ± 0.11 ^a^
7.5	-	80.92 ± 0.65 ^b^	27.23 ± 0.58 ^b^	3.24 ± 0.11 ^b^
10.0	-	74.33 ± 0.98 ^cd^	23.84 ± 0.37 ^de^	2.42 ± 0.17 ^de^
-	1.0	66.03 ± 0.83 ^f^	17.86 ± 0.13 ^i^	2.04 ± 0.10 ^f^
-	2.5	75.04 ± 0.83 ^cd^	20.68 ± 0.67 ^gh^	2.71 ± 0.14 ^cd^
-	5.0	80.34 ± 0.59 ^b^	26.48 ± 0.74 ^bc^	3.58 ± 0.16 ^a^
-	7.5	75.76 ± 0.92 ^c^	23.24 ± 0.37 ^ef^	2.91 ± 0.14 ^c^
-	10.0	70.44 ± 0.42 ^e^	19.67 ± 0.92 ^h^	2.08 ± 0.17 ^f^

Values are means ± SEM, n = 20 per treatment group. Means in a column without a common superscript letter differ (*p* < 0.05), as analyzed by one-way ANOVA and the Duncan test.

**Table 3:** Effect of auxins (IAA or IBA) with optimal concentrations of BA (5.0 μM) and Kin (5.0 μM) on shoot multiplication in callus mass obtained from internodal explants after 8 weeks of culture.

BA	Kin	NAA	IAA	IBA	Response %	Mean Number of Shoots	Mean Shoot Length (cm)
5.0	-	0.0	-	-	85.54 ± 0.58 ^d^	30.42 ± 0.51 ^jkl^	3.90 ± 0.11 ^bf^
5.0	-	0.5	-	-	90.22 ± 0.67 ^c^	41.23 ± 0.86 ^bc^	3.74 ± 0.11 ^defg^
5.0	-	1.0	-	-	98.44 ± 0.51 ^a^	54.63 ± 1.54 ^a^	4.54 ± 0.05 ^a^
5.0	-	1.5	-	-	92.48 ± 0.69 ^b^	42.84 ± 1.16 ^b^	4.26 ± 0.08 ^ab^
5.0	-	2.0	-	-	79.23 ± 0.62 ^ef^	28.47 ± 0.92 ^km^	3.58 ± 0.15 ^efh^
5.0	-	-	0.5	-	79.64 ± 0.51 ^ef^	33.42 ± 1.03 ^fi^	3.24 ± 0.18 ^hijk^
5.0	-	-	1.0	-	91.73 ± 0.71 ^bc^	43.67 ± 1.21 ^b^	4.32 ± 0.10 ^ab^
5.0	-	-	1.5	-	85.64 ± 0.62 ^d^	37.23 ± 1.24 ^de^	3.94 ± 0.27 ^be^
5.0	-	-	2.0	-	79.54 ± 0.69 ^ef^	27.81 ± 0.97 ^lm^	3.36 ± 0.18 ^ghij^
5.0	-	-	-	0.5	76.41 ± 0.89 ^gh^	30.25 ± 0.73 ^jkl^	3.04 ± 0.22 ^jl^
5.0	-	-	-	1.0	84.83 ± 0.66 ^d^	39.46 ± 0.51 ^cd^	4.04 ± 0.05 ^bd^
5.0	-	-	-	1.5	77.85 ± 0.86 ^fg^	31.81 ± 1.24 ^ghij^	2.96 ± 0.05 ^jl^
5.0	-	-	-	2.0	69.87 ± 0.86 ^ij^	26.81 ± 1.53 ^m^	2.78 ± 0.11 ^l^
-	5.0	0.5	-	-	79.67 ± 0.92 ^ef^	33.86 ± 0.86 ^fh^	3.57 ± 0.070 ^fi^
-	5.0	1.0	-	-	90.95 ± 0.71 ^c^	41.82 ± 1.28 ^bc^	4.37 ± 0.083 ^ab^
-	5.0	1.5	-	-	81.21 ± 0.86 ^e^	34.80 ± 1.16 ^ef^	3.94 ± 0.068 ^be^
-	5.0	2.0	-	-	75.57 ± 0.56 ^h^	30.83 ± 0.66 ^ikl^	3.48 ± 0.13 ^fi^
-	5.0	-	0.5	-	75.46 ± 0.47 ^h^	30.07 ± 0.83 ^jkl^	3.08 ± 0.12 ^il^
-	5.0	-	1.0	-	84.62 ± 0.68 ^d^	35.71 ± 0.70 ^ef^	4.18 ± 0.10 ^abc^
-	5.0	-	1.5	-	80.12 ± 0.63 ^e^	31.23 ± 0.58 ^hik^	3.84 ± 0.22 ^cdef^
-	5.0	-	2.0	-	70.76 ± 0.58 ^i^	28.68 ± 0.74 ^km^	3.12 ± 0.12 ^il^
-	5.0	-	-	0.5	70.36 ± 0.54 ^ij^	29.48 ± 0.67 ^jkm^	2.98 ± 0.14 ^jl^
-	5.0	-	-	1.0	81.29 ± 0.67 ^e^	34.61 ± 0.51 ^efg^	3.94 ± 0.12 ^be^
-	5.0	-	-	1.5	71.84 ± 1.04 ^i^	31.23 ± 0.66 ^hik^	2.94 ± 0.11 ^kl^
-	5.0	-	-	2.0	68.21 ± 1.10 ^j^	27.85 ± 1.16 ^lm^	2.74 ± 0.14 ^l^

Values are means ± SEM, n = 20 per treatment group. Means in a column without a common superscript letter differ (*p* < 0.05), as analyzed by one-way ANOVA and the Duncan test.

**Table 4:** Total polyphenols, flavonoids, tannins, and alkaloids in methanolic and ethanolic extracts from donor plant growing in soil in the botanical garden, ex-vitro-established micropropagated plants, and callus of *R. chalepensis*.

Phytochemicals	DP *	MP **	Callus ***
Total polyphenols (mg GAE/g DW)	94.75 ± 0.09 ^b^	143.92 ± 0.16 ^a^	53.11 ± 0.33 ^c^
Flavonoids (mg QE/g DW)	90.10 ± 0.57 ^b^	135.86 ± 0.87 ^a^	86.31 ± 0.61 ^c^
Tannins (mg TAE/g DW)	44.31 ± 1.07 ^b^	54.39 ± 0.09 ^a^	36.64 ± 0.85 ^c^
Alkaloids %	8.96 ± 0.16 ^b^	10.79 ± 0.21 ^a^	7.96 ± 0.23 ^c^

Values are means ± SEM, n = 3 per treatment group. * DP = donor plant growing in soil in the botanical garden; ** MP = ex-vitro-established micropropagated plants; *** callus = callus obtained on MS + 2,4-D (10 µM) + BA(1.0 µM). Means in a row without a common superscript letter differ (*p* < 0.05), as analyzed by one-way ANOVA and the Duncan test.

**Table 5:** Radical scavenging activity (%) of *R. chalepensis* donor plant growing in soil in the botanical garden, ex-vitro-established micropropagated plants, and callus obtained from MS + 2,4-D (10 µM) + BA (1.0 µM).

Concentrations (µg/mL)	DPPH Radical Scavenging Activity (%)		
	DP *	MP **	Callus ***
200	76.01 ± 0.11 ^e^	78.79 ± 0.03 ^e^	35.83 ± 0.16 ^d^
400	82.16 ± 0.02 ^c^	88.12 ± 0.02 ^b^	46.94 ± 0.05 ^e^
600	88.94 ± 0.09 ^a^	91.23 ± 0.12 ^a^	50.91 ± 0.14 ^c^
800	87.38 ± 0.27 ^b^	85.27 ± 0.02 ^c^	60.42 ± 0.22 ^b^
1000	80.41 ± 0.11 ^d^	82.33 ± 0.03 ^d^	72.91 ± 0.28 ^a^

Values are means ± SEM, n = 3 per treatment group. * DP = donor plant growing in soil in the botanical garden; ** MP = ex-vitro-established micropropagated plants; *** callus = callus obtained on MS + 2,4-D (10 µM) + BA(1.0 µM). Means in a row without a common superscript letter differ (*p* < 0.05), as analyzed by one-way ANOVA and the Duncan test.

**Table 6:** GC–MS analysis of *R. chalepensis* methanol extracts of the donor plant growing in soil in the botanical garden, ex-vitro-established micropropagated plants, and callus obtained from MS + 2,4-D (10 µM) + BA (1.0 µM).

S. No.	RT * (min)	Name of Compound	Area %			Molecular Weight (g/mol)	Structural Formula
			DP *	MP **	Callus ***		
1	9.303	1-Ethyl-2-pyrrolidinone	0.88	-	-	113.084	C_6_H_11_NO
2	17.767	4-(3,4-Methylenedioxyphenyl)-2-butanone	1.24	-	-	192.079	C_11_H_12_O_3_
3	20.082	2-(2-Hydroxyethoxy)phenol	1.21	-	-	154.063	C_8_H_10_O_3_
4	20.762	Oleyl Alcohol	0.99	-	-	268.277	C_18_H_36_O
5	23.027	7-Hydroxy-3-phenyl-2-thioxo-1,3-diazabicyclo(3.3.0)octan-4-one	4.65	-	-	248.062	C_12_H_12_N_2_O_2_S
6	24.444	p-Dimethylaminobenzylidene p-anisidine	21.55	-	-	254.142	C_16_H_18_N_2_O
7	26.894	Kokusaginine	24.22	-	11.75	259.084	C_14_H_13_NO_4_
8	28.043	1H-Indole,1-(trimethylsilyl)-5-[(trimethylsilyl)oxy]-	1.19	-	-	277.132	C_14_H_23_NOSi_2_
9	28.437	Podocarpa-8,11,13-trien-3-one, 13-isopropyl-12-methoxy-	40.74	27.60	-	314.225	C_21_H_30_O_2_
10	29.226	3-Phenylthiolane,S,S-dioxide	1.21	-	-	196.056	C_10_H_12_O_2_S
11	30.878	Arborinine	2.14	-	-	285.100	C_16_H_15_NO_4_
12	3.179	9,17-Octadecadienal, (Z)-	-	1.97	-	264.245	C_18_H_32_O
13	20.762	Bicyclo[3.1.1]heptane, 2,6,6-trimethyl-, (1.alpha.,2.beta.,5.alpha.)-	-	3.50	-	138.141	C_10_H_18_
14	21.047	Bicyclo[3.3.0]octan-3-one, 8-chloro-	-	1.09	-	158.05	C_8_H_11_ClO
15	21.257	Bicyclo[4.1.0]heptane, 3-methyl-	-	1.54	-	110.11	C_8_H_14_
16	22.213	2-Propenamide, N-cyclopropyl-3-(2-fluorophenyl)-, (E)-	-	1.26	-	205.09	C_12_H_12_FNO
17	23.043	3-Adamantan-1-yl-heptan-4-one	-	1.86	-	248.214	C_17_H_28_O
18	23.471	7H-Furo[3,2-g][1]benzopyran-7-one, 4-methoxy-	-	8.87	-	216.190	C_12_H_8_O_4_
19	23.664	Phytol	-	5.14	-	296.308	C_20_H_40_O
20	24.025	9,12,15-Octadecatrienoic acid, (Z,Z,Z)-	-	1.54	-	278.225	C_18_H_30_O_2_
21	24.1	2(1H)-Naphthalenone, octahydro-8a-hydroxy-4a-methyl-	-	1.95	-	182.131	C_11_H_18_O_2_
22	24.436	9H-Thioxanthen-9-one, 2-(1-methylethyl)-	-	38.72	-	254.077	C_16_H_14_OS
23	24.948	Trioxsalen	-	3.71	-	228.079	C_14_H_12_O_3_
24	31.893	dl-.alpha.-Tocopherol	-	1.26	-	430.381	C_29_H_50_O_2_
25	17.364	4-Methyl-2,5-dimethoxybenzaldehyde	-	-	4.22	180.079	C_10_H_12_O_3_
26	21.752	Hexadecanoic acid, methyl ester	-	-	2.60	270.256	C_17_H_34_O_2_
27	21.995	Benzenepropanoic acid, 3,5-bis(1,1-dimethylethyl)-4-hydroxy-, methyl ester	-	-	2.11	292.204	C_18_H_28_O_3_
28	22.238	Dictamnine	-	-	8.84	199.063	C_12_H_9_NO_2_
29	23.664	Methoxsalen	-	-	6.05	216.192	C_12_H_8_O_4_
30	24.444	2-Ethoxy-10H-acridin-9-one	-	-	46.84	239.095	C_15_H_13_NO_2_
31	25.015	2-(n-Propylethanimidoyl)-1H-indene-1,3(2H)-dione	-	-	3.36	229.11	C_14_H_15_NO_2_
32	25.199	7H-Furo[3,2-g][1]benzopyran-7-one, 4,9-dimethoxy-	-	-	1.22	246.053	C_13_H_10_O_5_
33	25.501	Naphtho[1,2-b]furan-2(3H)-one, 5-methyl-4-phenyl-	-	-	1.61	274.099	C_19_H_14_O_2_
34	25.787	2,11-Dimethyl-2,3,4,5,6,7-hexahydro-1H-2-benzazonine	-	-	1.48	203.167	C_14_H_21_N
35	26.558	Indole-5-carboxylic acid, 1,2,3-trimethyl-	-	-	2.20	203.095	C_12_H_13_NO_2_
36	28.463	Furo[2,3-b]pyridine-2-carboxylic acid, 6,7-dihydro-3-amino-4-methoxy-6-oxo-, phenyl ester	-	-	4.77	300.075	C_15_H_12_N_2_O_5_
37	29.243	trans-4-Dimethylamino-4’-methoxychalcone	-	-	2.96	281.142	C_18_H_19_NO_2_

* Rt—retention time; * DP = donor plant growing in soil in the botanical garden; ** MP = ex-vitro-established micropropagated plants; *** callus = callus obtained on MS + 2,4-D (10 µM) + BA(1.0 µM).

**Table 7:** Rutin and hesperidin content (µg/mg DW) in methanol extracts of *R. chalepensis*.

	Rutin (µg/mg DW)	Hesperidin (µg/mg DW)
DP *	31.03 ± 1.55 ^b^	91.44 ± 1.75 ^a^
MP **	55.90 ± 2.04 ^a^	93.30 ± 1.48 ^a^
Callus ***	21.60 ± 0.97 ^c^	59.11 ± 1.22 ^b^

* D = donor plant growing in soil in the botanical garden; ** MP = ex-vitro-established micropropagated plants; *** callus = callus obtained on MS + 2,4-D (10 µM) + BA(1.0 µM). Means in a column without a common superscript letter differ (*p* < 0.05), as analyzed by one-way ANOVA and the Duncan test

**Table 8:** Inter simple sequence repeat (ISSR) primers used to evaluate the genetic fidelity of ex-vitro-established micropropagated *R. chalepensis* plants.

Names of Primers	Sequence 5′–3′	Ta (°C) *	No. of Bands
UBC-825	ACA CAC ACA CAC ACA CT	46	14
UBC-827	ACA CAC ACA CAC ACA CG	50	16
UBC-834	AGA GAG AGA GAG AGA GYT	50	09
UBC-841	GAG AGA GAG AGA GAG AYC	50	14
UBC-855	ACA CAC ACA CAC ACA CYT	50	15
UBC-866	CTC CTC CTC CTC CTC CTC	55	13
UBC-868	GAA GAA GAA GAA GAA GAA	46	12
UBC-880	GGG TGG GGT GGG GTG	50	13
UBC-889	DBD ACA CAC ACA CAC ACA C	46	15
UBC-891	HVH TGT GTG TGT GTG TG	46	10
Total no. of bands			131
Average no. of bands /primers			13.1

* Ta: annealing temperature.

## Data Availability

Not applicable.
